# Analysis of bicycle helmet damage visibility for concussion-threshold impacts

**DOI:** 10.1080/23335432.2021.2014359

**Published:** 2021-12-17

**Authors:** Ana Cachau-Hansgardh, Caitlin McCleery, Manon Limousis-Gayda, Rami Hashish

**Affiliations:** National Biomechanics Institute, Santa Monica, United States

**Keywords:** Bicycle helmet testing, mTBI, Damage visibility, Material characterization, EPS

## Abstract

Any helmet involved in an accident should be replaced, regardless of appearance after impact. However, consumer compliance and interpretation of this recommendation is unclear, for which there is additional ambiguity for lesser impacts. This study aims to investigate the relation between helmet damage visibility and lesser impacts in line with concussion. As a preliminary model, a commercially available road-style helmet was chosen. Twelve helmets underwent impact attenuation testing; four were dropped from the standard testing height of 2 m, and eight from lower drop heights (0.34 and 0.42 m) associated with the production of linear accelerations (90 and 100 g, respectively) consistent with the production of concussion. Expanded polystyrene damage was assessed via flat punch penetration testing. American adults were then polled on helmet damage visibility based upon before and after photos. All helmets demonstrated damage to the expanded polystyrene liner in the form of altered material properties. Helmets dropped from 2 m displayed significant changes in elastic buckling (p < .01) and densification behavior (p < .01) as compared with lower drop height results. Adverse change in elastic buckling behavior was found to increase linearly with drop height (p < .001). Damage visibility was significant for helmets dropped from a 2-meter height, however, such a relation among the helmets impacted at the threshold for concussion was lacking. These findings suggest that for the chosen helmet model, consumers may be unable to distinguish between new helmets and helmets with diminished protective abilities.

## 1. Introduction

Since 1999, any bicycle helmet intended for sale within the United States must meet requirements set by the US Consumer Product Safety Commission (CPSC) (C.P.S.C 1998). One such requirement is for impact attenuation testing, where the CPSC requires exemplar helmets to withstand a drop from a 2-meter height onto a flat anvil. The helmet is in compliance if peak acceleration at impact does not exceed 300 g, a threshold associated with an 80% risk of skull fracture in forehead impacts (Mertz et al. 1997). Skull fracture due to occipital, side, and frontal flat plate impacts have furthermore been associated with peak anteroposterior accelerations of 375, 268, and 223 g, respectively (Yoganandan and Pintar 2004). Studies have found that due to this requirement, the effectiveness of current helmet design centers on preventing skull fracture and severe brain injuries rather than mild traumatic brain injuries (mTBI) (Bambach et al. 2013). Accordingly, the effects of less severe impacts, consistent with concussion production, are not as well documented.

Another requirement by the CPSC, as well as the equivalent Canadian regulatory body the CSA, is for distinct labelling stating that helmets are intended to be replaced after involvement in any moderate impact and should, by this standard, be considered single-use items (C.S.A 1989; C.P.S.C 1998). Bicycle helmet standards set by the European Union and the United Kingdom acknowledge that damage may not be visible (B.S.I 2012; C.E.N 2012). However, helmet labelling or packaging is not required to indicate this. Ultimately, judgment is left to the consumer, who may use the helmet appearance as an indicator for replacement regardless. For this reason, it is of interest to understand the relation between the extent of structural helmet damage and damage visibility as perceived by consumers.

One study assessed this relation through the collection of hospital data from which 10% of bicycle riders believed their damaged helmets were undamaged (Ching et al. 1997). As hospital visits are more likely with increase in injury severity, hospital data alone is unlikely to cover the full range of possible helmet damage, especially for lesser impacts. Another study took this into account by instead collecting helmets via a manufacturer’s return policy, although in this study damage visibility was not assessed (Smith et al. 1994). Accordingly, this study aims to better understand the relation between helmet damage and damage visibility for concussion-threshold impacts. To the knowledge of the authors, this relation has not been studied with the inclusion of the consumers viewpoint.

Several factors influence a consumer’s decision on when to replace their helmet. A fundamental consideration should be the helmet’s advised expiration date. However, these range considerably. For example, the helmet manufacturer Bell recommends 3 years, while the Snell Memorial Foundation suggests 5 years, and the CPSC 5 to 10 years Bell Product FAQ [WWW Document] 2019; Snell – FAQ [WWW Document] 2021; Which Helmet for Which Activity [WWW Document] 2014. Contrarily, studies have found helmet liners unchanged after up to 26 years (Kroeker et al. 2016; DeMarco et al. 2017). The decision for replacement is thus not a straightforward one. Clarification in other areas, such as the warning on damage visibility, could therefore help guide consumers.

**Figure 1.**
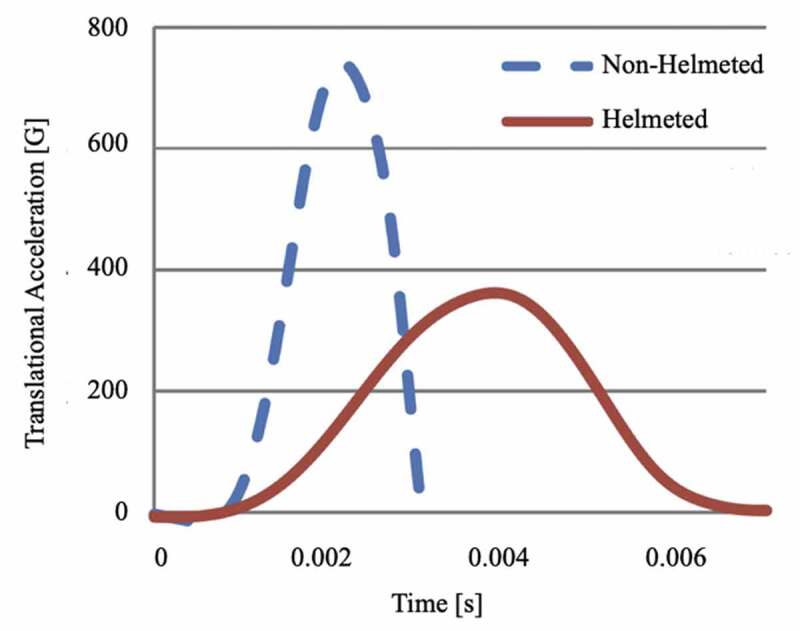
Impulse curves for helmeted and non-helmeted impacts in otherwise similar conditions (Levadnyi, Awrejcewicz, Finite Element Analysis of Impact for Helmeted and Non-helmeted Head 2018)

The protective abilities of bicycle helmets largely depend on the energy dissipating effects of the intermediate expanded polystyrene (EPS) layer. During impact, gradual compression of the EPS layer effectively lengthens impact duration, allowing for the dissipation of initial impact energy (Henderson 1995; Krundaeva et al. 2016; Bocciarelli et al. 2020). A comparison between helmeted and non-helmeted impulse curves over impact duration, as shown in Figure 1, demonstrates this energy transformation. While impulse remains constant for both, impact duration increases for the helmeted user, consequently decreasing peak acceleration delivered to the head (Levadnyi et al. 2018). The protective abilities of helmets primarily depend on two occurrences at impact: compression of the intermediate EPS layer and cracking of the outer polycarbonate shell. The decrease in energy transferred to the head is ultimately due to a portion of the impact energy converting into altered material properties or heat, given off as a by-product of rapid plastic deformation (Walker 2005; Bocciarelli et al. 2020). Altered material properties may take the form of increased material stiffness. A material that has undergone plastic deformation, or damage, may increase in stiffness via an increase in density (Krundaeva et al. 2016).

**Figure 2.**
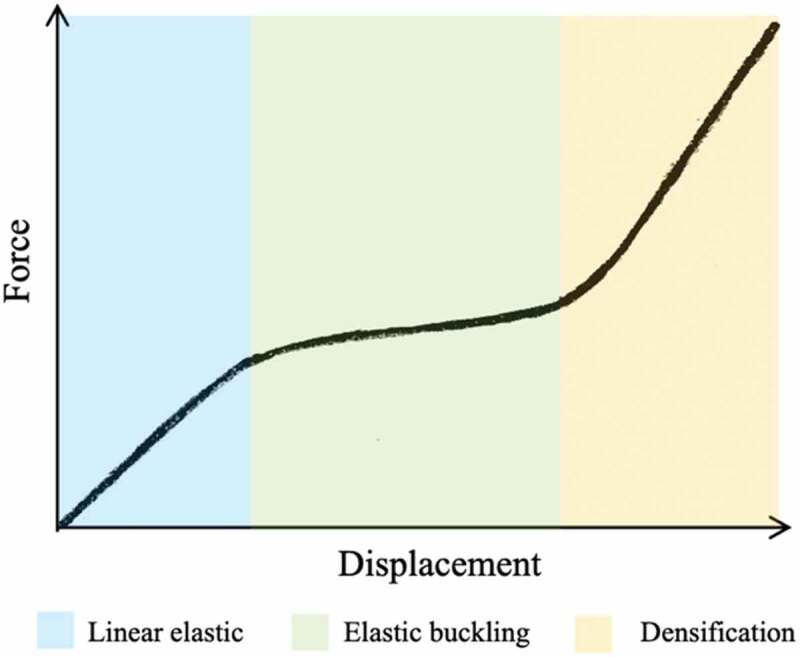
Typical foam polymer compression testing behavior

The force–displacement relation of foam polymers, such as EPS, under compression testing is expected to follow the behavior illustrated in Figure 2. The material initially exhibits linear elastic behavior until yield stress is reached, at which point the individual foam cells collapse and elastic buckling occurs. Finally, material densification begins when the majority of the cells have collapsed, resulting in a rapid stress increase (Gibson and Ashby 1997).

## 2. Materials & Methods

Exemplar helmets of the same model were evaluated for sustained damage in three steps. First, twelve helmets underwent impact attenuation testing. Test specimens were then taken from the helmets and experimentally tested for altered material properties. American adults were then polled on helmet appearance before and after impact to evaluate the visibility of damage. In addition, cross-sectional microscopic images were taken of the test specimens. Prerequisites to testing included defining a threshold correlated with concussion and a basis for helmet selection.

### Concussion criteria

Universal agreement on what constitutes a concussion, a form of mTBI, has long challenged the scientific community. Many definitions build on reported symptoms such as the one proposed by the American Orthopaedic Society for Sports Medicine Concussion Workshop Group: ‘Any alteration in cerebral function caused by a direct or indirect (rotation) force transmitted to the head resulting in one or more of the following acute signs or symptoms: a brief loss of consciousness, light-headedness, vertigo, cognitive and memory dysfunction, tinnitus, blurred vision, difficulty concentrating, amnesia, headache, nausea, vomiting, photophobia, or a balance disturbance’. (Wojtys et al. 1999). However, a purely quantitative definition is required for helmet design and experimentation purposes, such as an injury threshold based upon peak linear acceleration.

An injury threshold of 80 to 90 g for linear acceleration has been proposed for impact durations exceeding 4 ms (Gurdjian 1972). Likewise, another study found peak accelerations greater than 90 g likely to produce mTBI, however linear accelerations of 80 g were found non-injurious (Gurdjian et al. 1964). A 90 g mTBI threshold was also proposed for impacts longer than 9 ms (Ono and Kanno 1996). A separate study using a finite element brain injury model based upon head-to-head collision data taken from football games predicted a 25%, 50%, and 80% probability of mTBI for resultant linear accelerations of 66 g, 82 g, and 106 g, respectively (Zhang et al. 2004). Two further studies on professional athletes found similar average peak accelerations of 98 g and 98.68 g, respectively (Pellman et al. 2003; Brennan et al. 2017). Using these findings, lower and upper bounds to the threshold for concussion were determined. A lower bound of 90 g was chosen as the value reappears frequently without speculation, and the upper bound was set to 100 g for an interval that encompasses the majority of the reported thresholds and excludes outliers.

Two other commonly used metrics for measuring mTBI are severity index (SI) and head injury criterion (HIC). The SI accounts for resultant translational acceleration over time, as illustrated in Figure 1, and is calculated according to Equation 1.

(1) $$SI=Ta{\left(t\right)}^{2.5}dt$$

Where *T* is the acceleration pulse duration, and *a(t)* is the resultant linear acceleration measured at the headform’s center of gravity (Schmitt et al. 2010). Meanwhile HIC, a modified version of the SI, is calculated according to Equation 2.

(2) $$HIC=max{\left[\frac{1}{t_{2}-t_{1}}\overset{t_{2}}{\underset{t_{1}}{\int }}a\left(t\right)dt\right]}^{2.5}\left(t_{2}-t_{1}\right)$$

**Figure 3.**
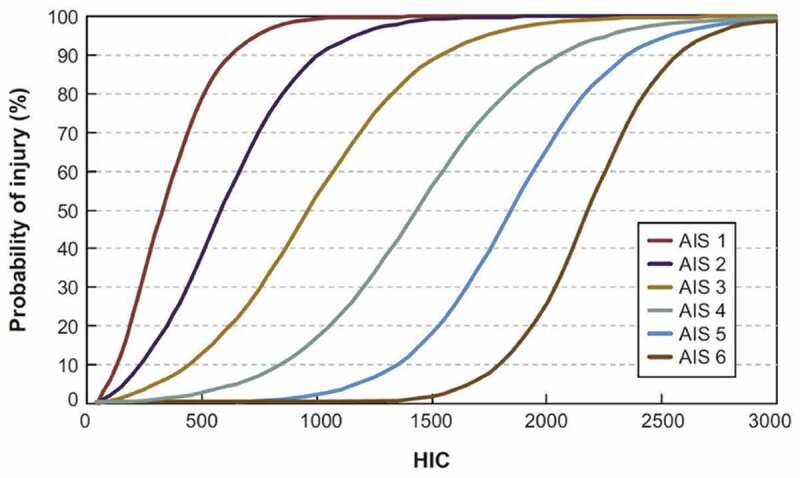
Correlation between HIC value and AIS scale (Moure-Guardiola, Rubio Diaz, Evaluation of Combat Helmet Behavior under Blunt Impact 2020)

Where integration is instead taken between the start and end times, *t*_1_ and *t*_2_, which produce a maximum value within a set time difference. HIC15, for example, utilizes a 15 ms time interval. Significance in HIC values can be derived from the AIS injury severity scale, as shown in Figure 3. Mild concussion without loss of consciousness coincides with AIS level 1, while loss of consciousness up to one hour coincides with AIS level 2 (Chybowski and Przetakiewicz 2020). Minor and major skull fracture coincide with AIS level 2 and 3, respectively (Shelley 2016).

### Helmet selection

While results from this study cannot account for the variability between bicycle helmets, the helmet selection was still of significance. Three criteria were defined in order to facilitate the study timeline and increase likelihood of choosing a model representative of more commonly used helmets.

- **Road style helmet**: Road style helmets are most frequently used by everyday bicyclists (Bicycle Helmet Saf. Inst., 2020). These helmets have an elongated shape with vents, an intermediate EPS layer, and a polycarbonate shell. They also tend to perform better than rounder commuter helmets (IIHS HLDI, 2018).

- **Simple design**: Helmets with more advanced systems, such as MIPS, were excluded to simplify the characterization of the material behavior responsible for helmet protective abilities.
- **High rating**: Of the remaining helmets, the highest rated was then chosen. The non-profit product-testing organization Consumer Reports was used to compare ratings.

All helmets had the same blue two-toned pattern to better facilitate comparison.

### 2.1. Impact Attenuation Testing

The procedure was conducted according to CPSC regulations for the standard 2 m drop height as well as for two lower drop heights conducive to concussion (C.P.S.C 1998). The procedure is detailed below.

#### Preparation

*Calibration*: Resultant acceleration during impact is dependent on helmet drop height. Calibration of the appropriate drop heights, which would produce a 90 g and 100 g peak acceleration, was first conducted using three exemplar helmets. To attain data in the appropriate range, the drop heights used for calibration were first approximated.

Research suggests that helmet impact velocity is correlated with helmet thickness, for which an estimated helmet thickness of 28 to 30 mm in the chosen helmet model coincides with a velocity of approximately 3.5 m/s and 3.9 m/s for a 90 g and 100 g peak acceleration, respectively (DeMarco et al. 2016). Corresponding drop heights were calculated to 0.64 m and 0.77 m. To account for variability between helmets and increase the span of data, the three drop heights were set to 0.5, 0.75, and 1 m. Finally, they were increased by 5 cm to account for friction in the drop rig, an adjustment used in previous studies and recommended by the CPSC (C.P.S.C 1998; Cripton et al. 2014). The final drop heights were set at 0.55, 0.8, and 1.05 m.

**Figure 4.**
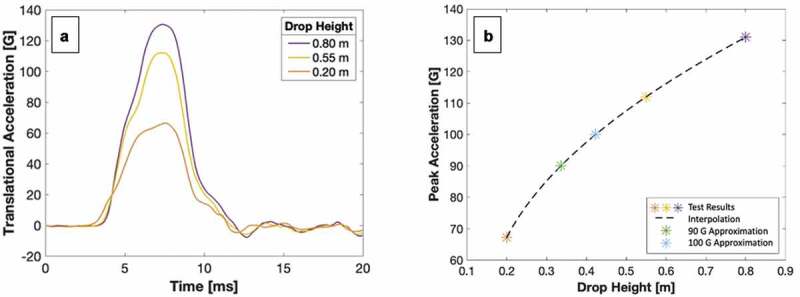
Calibration of drop heights conducive to concussion using three trial helmets. A – Translational acceleration over impact duration. B – Interpolation of trials

The first two tests completed at 0.8 m and 0.55 m produced peak accelerations at 131 g and 112 g, respectively. As both peak accelerations were substantially higher than the upper threshold for concussion of 100 g, the drop height of the third test was recalibrated. Initial interpolation of the first two results indicated that a drop height of approximately 0.26 m would produce a peak acceleration at the lower threshold for concussion of 90 g. To minimize the risk of the sought-after drop heights being outside of the tested range, the third test was set to 0.2 m, which produced a peak acceleration of 67.2 g. A cubic spline interpolation of the three tests produced test drop heights of 0.34 m and 0.42 m, for a 90 g and 100 g peak acceleration, respectively. Results are illustrated in Figure 4.

#### Pre-conditioning

Each helmet was placed in one of the following conditioning environments before testing and then tested within 2 minutes of removal as per CPSC requirements (C.P.S.C 1998). Fifteen helmets were conditioned in total: three for each of the four conditioning environments, with an additional three ambient-conditioned helmets for calibration.

- **Ambient environment**: 17°C to 27°C (63 to 81°F), and 20% to 80% relative humidity for at least 4 hours before testing.
- **Wet environment**: Immersed in potable water at a temperature of 17 to 27 °C (63 to 81 °F) for 4 to 24 hours before testing.
- **Low temperature**: −17°C to −13°C (1 to 9°F) for 4 to 24 hours before testing.
- **High temperature**: 47°C to 53°C (117 to 127°F) for 4 to 24 hours before testing.

#### Testing procedure

**Figure 5.**
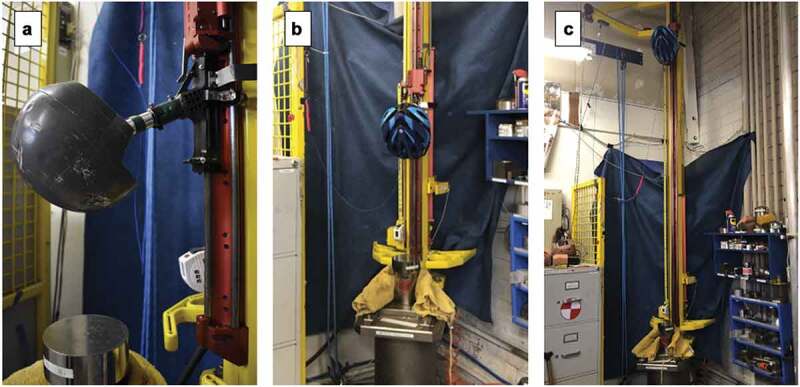
Impact attenuation testing monorail and test fixture with magnesium ISO J headform. A – Headform mount angle. B – Exemplar helmet mounted at one of the lower drop heights. C – Exemplar helmet mounted at the standard 2 m drop height

Impact attenuation testing followed the procedure in line with CPSC requirements, as outlined below (C.P.S.C 1998). All helmets were photographed directly before and directly after testing. The testing set up is shown in Figure 5.

1. Position the helmet on a low-resonance magnesium headform (sized to the sample helmet) with the brow parallel to the basic plane.
2. Tighten the retention strap to secure the helmet to the headform.
3. Orient the headform on the monorail test fixture so that the headform’s vertical axis points downward and 45 degrees to the direction of gravity.
4. Raise the test fixture to the helmet’s assigned drop height.
5. Release the test fixture onto a flat anvil with an impact face having a minimum diameter of 125 mm (4.92 in) and at least 24 mm (0.94 in) in thickness.
6. Measure impact velocity for the last 40 mm (1.57 in) of free-fall.
7. Record the maximum acceleration during impact.

**Figure 6.**
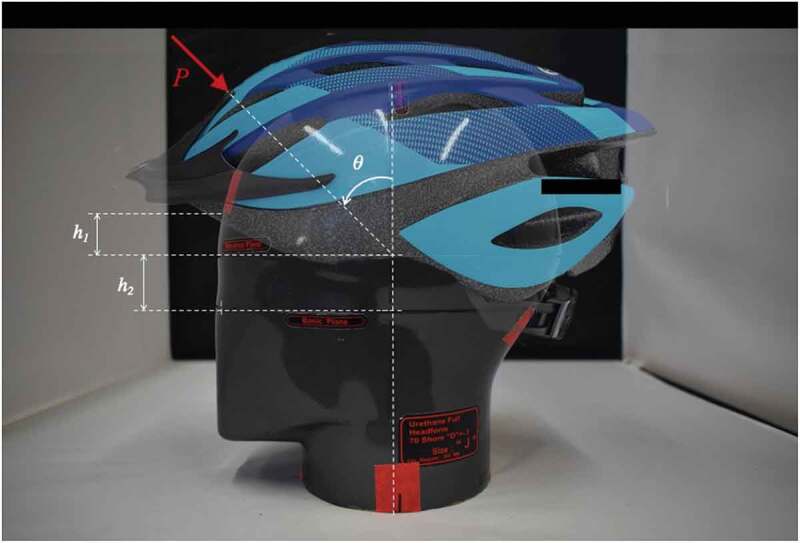
Magnesium ISO-J headform dimensions with impact attenuation testing parameters and projection of the chosen helmet model

The chosen headform was an ISO J magnesium model with parameters listed in Table 1. The mounting angle, *θ*, was set to 45°, for a center of impact (COI) along the helmet’s line of symmetry in line with impact force, *P*, as depicted in Figure 6.

**Table 1.** Impact attenuation testing parameters involving the magnesium ISO J headform

Description	Parameter	Value
Reference plane to helmet distance	h_1_	24 mm
Basic- to reference plane distance	h_2_	27.5 mm
Mount angle	θ	45°
Circumference	-	577 mm
Mass	-	4.7 kg

### 2.2. Material Testing

Material damage can be defined as plastic deformation caused by stresses exceeding the material’s yield strength, resulting in locally altered material properties.

Experimentally testing for alteration in EPS material properties can reveal if damage has been sustained. As helmets’ functionality lies in their ability to be crushed, compression testing was the chosen testing method (Henderson 1995). Compression testing is also beneficial with regard to the complex geometry of the chosen helmet model. Test specimen preparation first involved removing the rear half of the helmet, the outer polycarbonate shell, and the comfort liner. In order to prevent undesired influence of material properties, the specimen size was not further reduced (e.g. thermal effects from heating while cutting).

**Figure 7.**
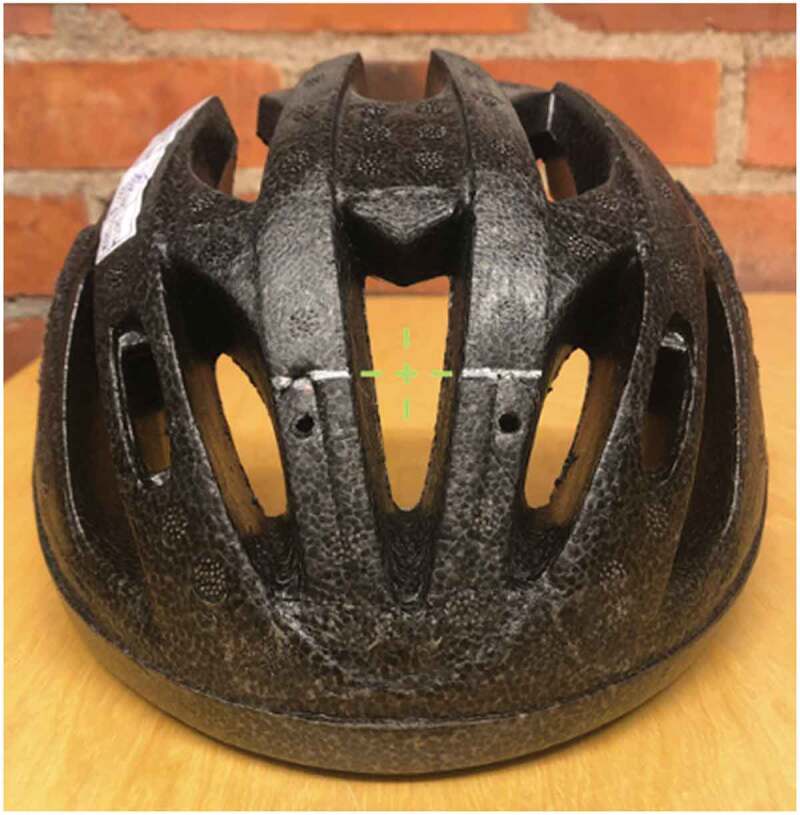
Center of impact projection and testing sites on a helmet test specimen

Testing sites nearest to the COI were then identified and marked. The COI was in line with the central vent, effectively making the first points of contact between the helmet and the anvil along the two slats framing the central vent as depicted in Figure 7.

**Figure 8.**
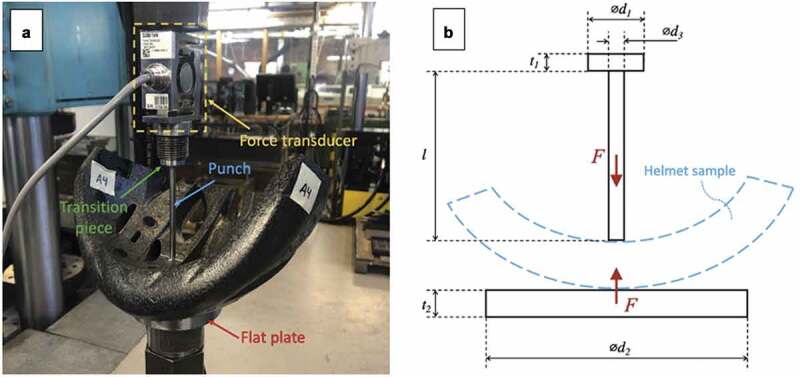
Lab set up. A – Helmet test specimen. B – Geometry illustration

The testing technique ultimately chosen was a quasi-static flat punch penetration test. Set up is shown in Figure 8 and dimensions are listed in Table 2. To minimize undesired bending and to promote isolated compression behavior, the concave and convex faces of the test specimen were oriented to be in contact with the punch and plate, respectively.

**Table 2.** Flat punch penetration test dimensions. All units in millimeters

Description	Dimension	Value
Transition piece thickness	⊘ d_1_	20
Transition piece thickness	t_1_	5
Punch diameter	⊘ d_3_	5
Punch length	l	80
Flat plate diameter	⊘ d_2_	80
Flat plate thickness	t_2_	13

Testing was performed on a servo-hydraulic testing machine, the input for which can be found in Table 3. Due to the relatively low stiffness of EPS, the preload was set to a nominal amount, and a force transducer with a nominal load of 1 kN was utilized to better record the lower forces. Inertial effects by the ramp rate are considered negligible, qualifying the test as quasi-static.

**Table 3.** Servo-hydraulic testing machine input variables

Variable	Value
Ramp rate	0.05 mm/s
Ramp end level	−25 mm
Time between samples	0.20 s

### 2.3. Damage Visibility

American adults were surveyed to determine the extent of damage visibility in each impacted helmet. Each respondent was asked to judge before and after photos taken of the outer forward-facing portion of the helmet where contact was made with the anvil during impact attenuation testing. Each photo was prompted by the question ‘Examine the following helmet. Does the helmet show visible damage?’, to which respondents could answer ‘Yes’, ‘No’, or ‘Unsure’.

Question and answer order was randomized to improve validity. Respondent selection was randomized through random device engagement, resulting in a range of respondents from 18 to 69 years old, of which 40% were female (Rothschild and Konitzer 2020). The survey was circulated via the polling site *Pollfish.com* to a total of 151 respondents for a 95% confidence interval and an 8% margin of error. This methodology is common in assessing the subjective opinion of regular consumers (Rodiek and Fried 2005; Mantiuk et al. 2012; Cortesão et al. 2020).

### 2.4. STATISTICAL ANALYSIS

Impact attenuation- and material testing data were assessed for statistical significance through a two-way analysis of variance (ANOVA) test for the variables drop height and conditioning environment. Post hoc testing was conducted using the Tukey Honest Significant Difference (HSD) test.

Damage visibility data was assessed for statistical significance through three Chi-square tests for independence which were formatted as follows:

- Response to damage visibility (Yes, No, Unsure) to helmet drop height (0.34 m, 0.42 m, 2 m)
- Response to damage visibility (Yes, No, Unsure) to conditioning environment (Ambient, Wet, Cold, Hot)
- Response to damage visibility (Yes, No) to individual helmets’ appearance before and after testing (Before, After).

## 3. Results

### 3.1. Impact Attenuation Testing

**Figure 9.**
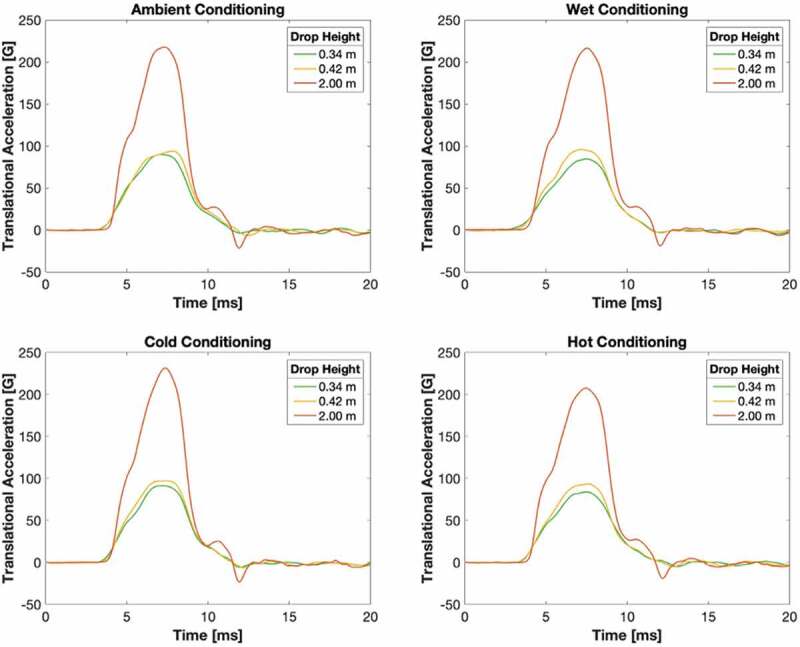
Translational acceleration over impact duration, subdivided by conditioning environment

Translational acceleration over impact duration for the bicycle helmets dropped from the standard 2 m height and the two calibrated concussion-threshold drop heights were recorded and are shown in Figure 9. Recorded data are presented in Table 4.

**Table 4.** Impact attenuation testing HIC15, SI, energy and velocity values by helmet drop height and conditioning environment

		Conditioning					
Parameter	Drop height	Ambient	Wet	Cold	Hot	M	SD
Peak acceleration [g]	0.34 m	90.3	84.8	91.8	84.3	87.8	3.30
	0.42 m	94.3	96.3	97.3	93.8	95.4	1.43
	2 m	218	217	233	206	219	9.60
HIC	0.34 m	187	165	194	168	179	12.3
	0.42 m	226	228	240	221	229	6.98
	2 m	1468	1474	1551	1410	1476	50.1
SI	0.34 m	216	187	223	191	204	15.5
	0.42 m	253	259	274	249	259	9.50
	2 m	1771	1699	1876	1624	1743	93.0
Energy [J]	0.34 m	15	14.9	15	15.1	15	0.07
	0.42 m	18.3	18.4	18.3	18.3	18.3	0.04
	2 m	89.4	90	89.7	89.5	89.7	0.23
velocity [m/s]	0.34 m	2.47	2.47	2.47	2.48	2.47	0.00
	0.42 m	2.73	2.74	2.73	2.73	2.73	0.00
	2 m	6.04	6.06	6.04	6.04	6.05	0.01

Note. M indicates mean. SD indicates standard deviation.

#### Peak acceleration

There was a statistically significant difference in peak acceleration by drop height (F(2,11) = 742, p < .001) and a non-significant difference between conditioning environments (F(3,11) = 2.83, p = .13) as determined by a two-way ANOVA.

#### HIC15

There was a statistically significant difference in HIC15 by drop height (F(2,11) = 2620, p < .001) and a non-significant difference between conditioning environments (F(3,11) = 2.39, p = .17) as determined by a two-way ANOVA.

#### SI

There was a statistically significant difference in SI by drop height (F(2,11) = 1000, p < .001) and a non-significant difference between conditioning environments (F(3,11) = 1.93, p = .23) as determined by a two-way ANOVA.

#### Energy

There was a statistically significant difference in energy by drop height (F(2,11) = 244,000, p < .001) and a non-significant difference between conditioning environments (F(3,11) = 0.714, p = .58) as determined by a two-way ANOVA.

#### Velocity

There was a statistically significant difference in velocity by drop height (F(2,11) = 381,000, p < .001) and a non-significant difference between conditioning environments (F(3,11) = 1.60, p = .29) as determined by a two-way ANOVA.

Examining the results by drop height, post hoc comparisons using the Tukey HSD test revealed that peak acceleration, HIC15, SI, energy, and velocity were significantly lower in the 0.34 m group (p < .01) and the 0.42 m group (p < .01) compared with the 2 m group. Energy and velocity were also significantly lower in the 0.34 m group compared with the 0.42 m group (p < .01). For peak acceleration, HIC15 and SI, the difference between the 0.34 m and 0.42 m groups was non-significant.

Interaction between drop height and conditioning environment was non-significant for peak acceleration, HIC15, SI, energy, and velocity.

### 3.2. Material Testing

**Figure 10.**
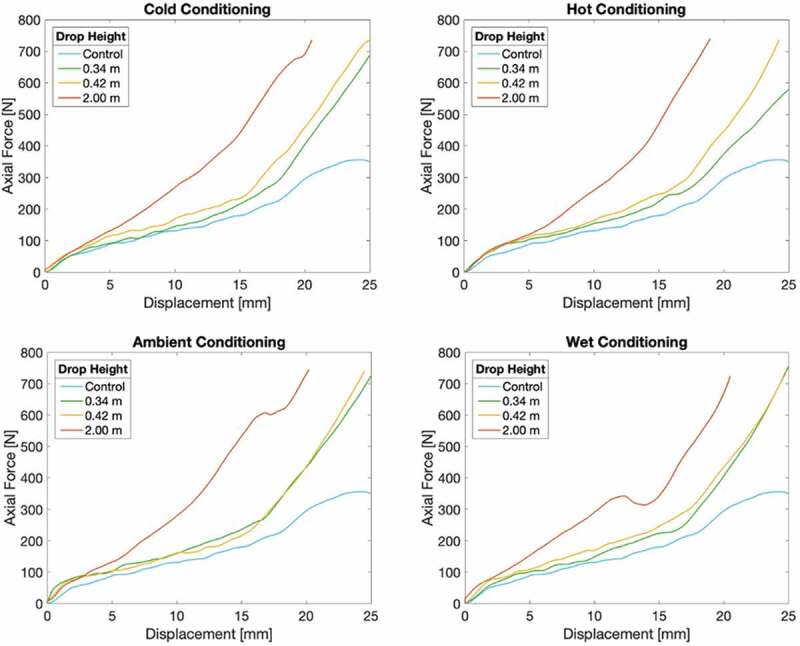
Axial force over punch displacement for test specimen taken from impacted helmets in comparison with a non-impacted control helmet grouped by conditioning environment

Force by punch displacement data for the impacted helmet specimens with a non-impacted control helmet is presented in Figure 10. The approximated slopes of the three material behavior zones are compiled in Table 5. The control helmet produced an approximated slope of 32.9, 9.19, and 34.6 N/mm in the linear elastic, elastic buckling, and densification zones, respectively.

**Table 5.** Average characteristic material behavior slopes as estimated based upon material testing results

		Conditioning					
Material behavior slope	Drop height	Ambient	Wet	Cold	Hot	M	SD
Linear elastic	0.34 m	74.7	30.4	32.4	42.1	44.9	17.8
	0.42 m	47.0	52.3	55.4	55.8	52.6	3.53
	2 m	46.7	61.1	46.2	45.6	49.9	6.47
Elastic buckling	0.34 m	10.2	9.39	10.3	10.3	10.0	0.37
	0.42 m	11.7	12.6	10.1	10.8	11.3	0.94
	2 m	30.0	27.3	27.6	29.0	28.5	1.08
Densification	0.34 m	52.6	50.7	57.7	42.1	50.8	5.63
	0.42 m	47.7	52.3	42.9	55.8	49.7	4.87
	2 m	59.9	61.1	62.1	65.7	62.2	2.17

Note. All measurements in N/mm. M indicates mean. SD indicates standard deviation.

#### Linear elastic slope

The difference between the slope of the linear elastic region was non-significant when examining the data by drop height (F(2,11) = 0.291, p = .76) or by conditioning environment (F(3,11) = 0.339, p = .80) as determined by a two-way ANOVA.

#### Elastic buckling slope

There was a statistically significant difference in the slope of the elastic buckling region by drop height (F(2,11) = 411, p < .001) and a non-significant difference between conditioning environments (F(3,11) = 0.832, p = .52) as determined by a two-way ANOVA.

Post hoc comparisons using the Tukey HSD test revealed that the elastic buckling slope was significantly lower in the 0.34 m group (p < .01) and the 0.42 m group (p < .01) compared to the 2 m group. The difference between the 0.34 m and 0.42 m groups was non-significant.

**Figure 11.**
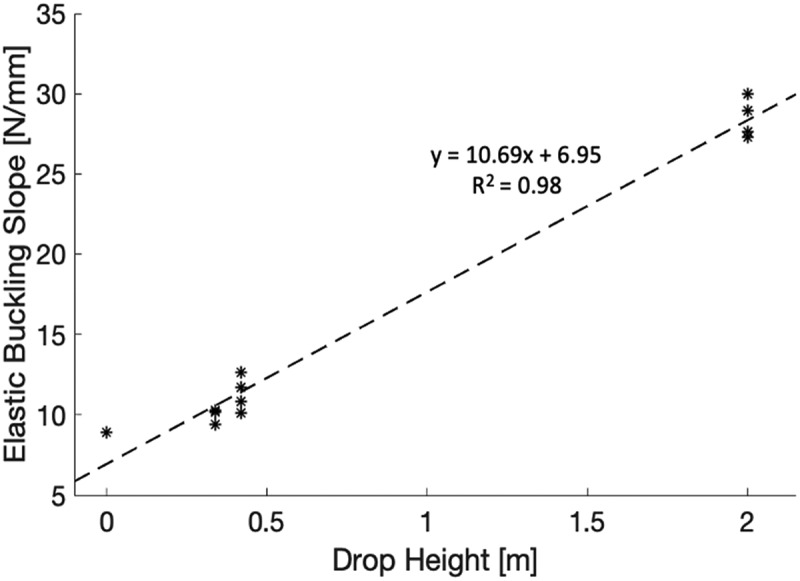
Relation between approximated elastic buckling slope of 13 helmet test specimens at COI and helmet drop height

A simple linear regression was calculated using data collected from the conditioned helmets in addition to the control helmet data to assess whether drop height predicts elastic buckling slope, see Figure 11. Results indicated that drop height explained 98% of variance in elastic buckling slope (R^2^^ ^= .98, F(1,11) = 649, p < .001). It was found that drop height significantly predicted the elastic buckling slope, (β = 0.99, t = 25.5, p <.001).

#### Densification slope

There was a statistical trend in difference between densification slope by drop height (F(2,11) = 4.85, p = .056) and a non-significant difference between conditioning environments (F(3,11) = 0.025, p = .99) as determined by a two-way ANOVA.

Post hoc comparisons using the Tukey HSD test revealed that the densification slope was significantly lower in the 0.34 m group (p < .05) and the 0.42 m group (p < .05) compared to the 2 m group. The difference between the 0.34 m and 0.42 m groups was non-significant.

#### Change in helmet thickness

**Figure 12.**
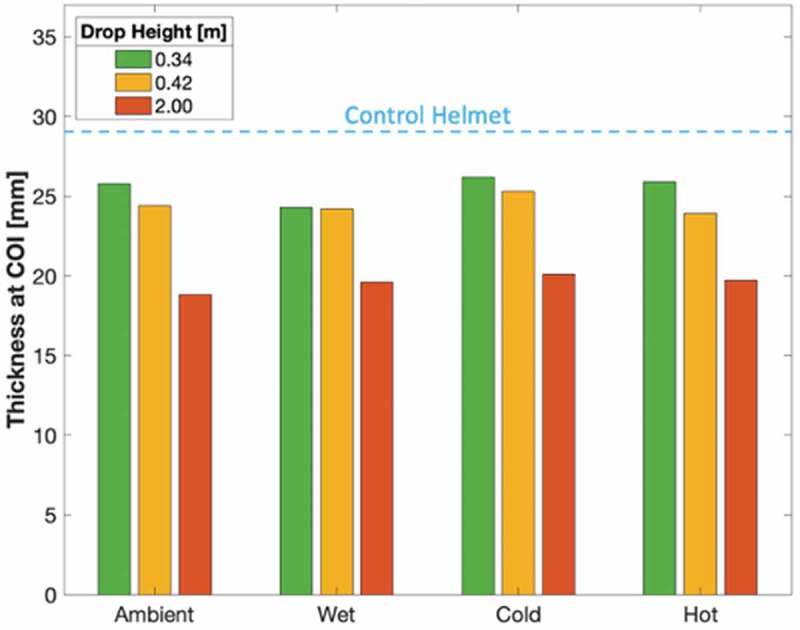
Estimated helmet thickness after impact based on flat punch penetration testing initial loading position

Helmet thickness at the COI was estimated by extracting the initial distance between punch and plate during material testing. The change in helmet thickness was approximated to the values in Table 6 by comparing final thickness with the thickness of the control helmet, of 29.0 mm, as depicted in blue in Figure 12.

**Table 6.** Estimated change in helmet thickness based upon flat punch penetration testing initial loading position and control helmet thickness at center of impact at 29.0 mm

	Conditioning					
Drop height	Ambient	Wet	Cold	Hot	M	SD
0.34 m	3.20	4.70	2.80	3.10	3.45	0.74
0.42 m	4.60	4.80	3.70	5.10	4.55	0.52
2 m	10.2	9.40	8.90	9.30	9.45	0.47

*Note*. All measurements in mm. M indicates mean. SD indicates standard deviation.

There was a statistically significant difference in the change in helmet thickness by drop height (F(2,11) = 126, p < .001) and a non-significant difference between conditioning environments (F(3,11) = 2.26, p = .18) as determined by a two-way ANOVA.

Post hoc comparisons using the Tukey HSD test revealed that the change in helmet thickness was significantly lower in the 0.34 m group (p < .01) and the 0.42 m group (p < .01) compared to the 2 m group. The difference between the 0.34 m and 0.42 m groups was non-significant.

Interaction between drop height and conditioning environment was non-significant for the linear elastic, elastic buckling, and densification zones, as well as for change in helmet thickness.

**Figure 13.**
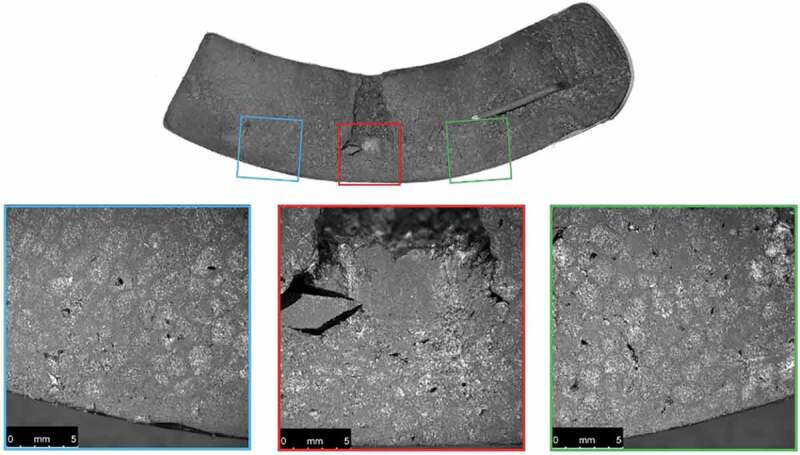
Microscopic images of a cross-section of the control helmet near the COI after material testing

Cross-sections of the ambient-conditioned test specimens were also photographed after material testing as shown in Figures 13 through 16.

**Figure 14.**
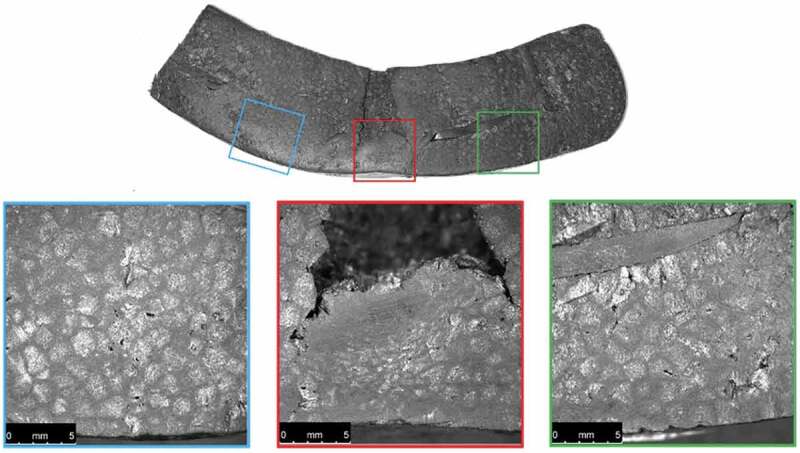
Microscopic images of a cross-section of the ambient-conditioned helmet dropped from 0.34 m near the COI after material testing

**Figure 15.**
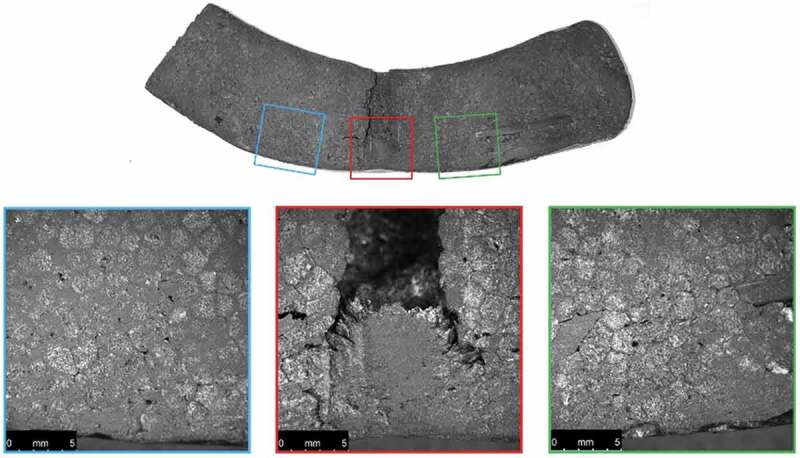
Microscopic images of a cross-section of the ambient-conditioned helmet dropped from 0.42 m near the COI after material testing

**Figure 16.**
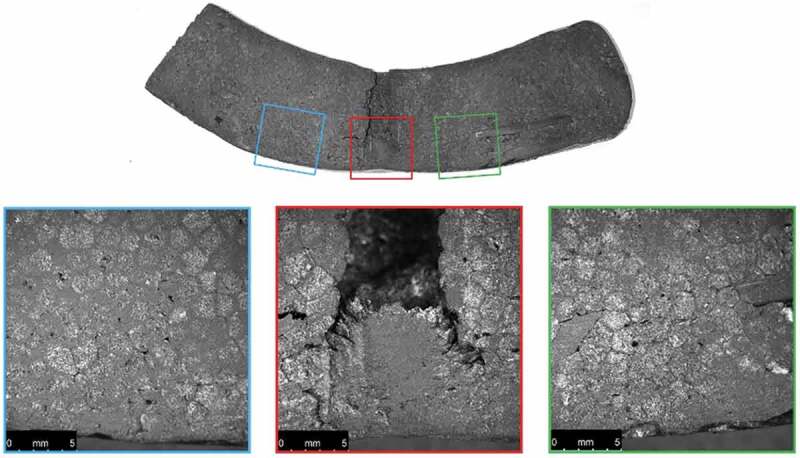
Microscopic images of a cross-section of the ambient-conditioned helmet dropped from 2 m near the COI after material testing

### 3.3. Damage Visibility

Results are listed in (Table (7,8)).

**Table 7.** Comparison in response to damage visibility to drop height with results grouped by conditioning environment

		Drop height				
Conditioning	Damage visibility response	0.34 m	0.42 m	2 m	Total	Chi square tests of independence
Ambient	Yes	45(−1.4)	33(−3.9)*	77(5.3)*	155	χ^2^ (4) = 34.74 p < .001 φ = 0.28 n = 453 (z = ± 2.77)
	No	92(2.4)	91(2.2)	57(−4.6)*	240	
	Unsure	14(−1.6)	27(2.3)	17(−0.7)	58	
Wet	Yes	50(−1.0)	43(−2.5)	72(3.5)*	165	χ^2^ (4) = 13.26 p = .01 φ = 0.17 n = 453 (z = ± 2.77)
	No	84(1.1)	88(1.9)	64(−2.9)*	236	
	Unsure	17(−0.1)	20(0.8)	15(−0.7)	52	
Cold	Yes	44(−1.9)	49(−0.9)	67(2.8)*	160	χ^2^ (4) = 11.99 p = .02 φ = 0.16 n = 453 (z = ± 2.77)
	No	93(2.2)	88(1.2)	65(−3.4)*	246	
	Unsure	14(−0.5)	14(−0.5)	19(1.1)	47	
Hot	Yes	49(−1.7)	33(−5.0)*	90(6.7)*	172	χ^2^ (4) = 54.36 p < .001 φ = 0.35 n = 453 (z = ± 2.77)
	No	82(1.0)	104(5.4)*	45(−6.4)*	231	
	Unsure	20(1.1)	14(−0.8)	16(−0.2)	50	

Note. Adjusted standardized residuals appear in parentheses below group frequencies. φ = effect size. z = z-criteria corresponding to p < .05 after Bonferroni adjustment. * = p < .05.

**Table 8.** Comparison in response to damage visibility to conditioning environment with results grouped by drop height

		Conditioning					
Drop height	Damage visibility response	Ambient	Wet	Cold	Hot	Total	Chi square tests of independence
0.34 m	Yes	45(−0.4)	50(0.6)	44(−0.6)	49(0.4)	188	χ^2^ (6) = 3.13 p = .79 φ = 0.07 n = 604 (z = ± 2.87)
	No	92(0.8)	84(−0.7)	93(1.0)	82(−1.1)	351	
	Unsure	14(−0.7)	17(0.2)	14(−0.7)	20(1.1)	65	
0.42 m	Yes	33(−1.4)	43(0.7)	49(2.0)	33(−1.4)	158	χ^2^ (6) = 12.74 p = .05 φ = 0.15 n = 604 (z = ± 2.87)
	No	91(−0.3)	88(−0.9)	88(−0.9)	104(2.2)	371	
	Unsure	27(2.4)	20(0.4)	14(−1.4)	14(−1.4)	75	
2 m	Yes	77(0.1)	72(−0.8)	67(−1.8)	90(2.5)	306	χ^2^ (6) = 8.76 p = .19 φ = 0.12 n = 604 (z = ± 2.87)
	No	57(−0.1)	64(1.2)	65(1.4)	45(−2.5)	231	
	Unsure	17(0.1)	15(−0.5)	19(0.7)	16(−0.2)	67	

Note. Adjusted standardized residuals appear in parentheses below group frequencies. φ = effect size. z = z-criteria corresponding to p < .05 after Bonferroni adjustment.

A significant relation was found between response to damage visibility and drop height within each group of similarly conditioned helmets, see Table 7. Post hoc comparisons of the adjusted residuals revealed that helmets dropped from 2 m had a significantly higher rate in respondents answering ‘Yes’ regarding damage visibility and a significantly lower rate of respondents answering ‘No’ within all four conditioning groups. In addition, ambient- and hot-conditioned helmets dropped from 0.42 m received significantly fewer respondents answering ‘Yes’. Hot-conditioned helmets dropped from 0.42 m also received a significantly higher rate in respondents answering ‘No’. No other relations were significantly associated.

The relation between the response to damage visibility and conditioning environment was significant among helmets dropped from 0.42 m and insignificant among helmets dropped from 0.34 m and 2 m, see Table 8. However, post hoc comparisons of the adjusted residuals did not present any significant associations.

Results are listed in Tables 9–11.

**Table 9.** Comparison in response to damage visibility and helmet appearance before and after impact attenuation testing. Results for helmets dropped from 0.34 m

Conditioning	Damage visibility response	Drop height: 0.34 m			Chi-square tests of independence
		Before	After	Total	
Ambient	Yes	37	45	82	χ^2^ (1) = 0.74 p = .39 φ = 0.05 n = 269
	No	95	92	187	
Wet	Yes	46	50	96	χ^2^ (1) = 0.11 p = .74 φ = 0.02 n = 264
	No	84	84	168	
Cold	Yes	35	44	79	χ^2^ (1) = 1.10 p = .29 φ = 0.06 n = 270
	No	98	93	191	
Hot	Yes	32	49	81	χ^2^ (1) = 6.83 p = .01 φ = .16 n = 271
	No	108	82	190	

Note. φ = effect size. Respondents answering ‘Unsure’ were excluded.

**Table 10.** Comparison in response to damage visibility and helmet appearance before and after impact attenuation testing. Results for helmets dropped from 0.42 m

Conditioning	Damage visibility response	Drop height: 0.42 m			Chi-square tests of independence
		Before	After	Total	
Ambient	Yes	35	33	68	χ^2^ (1) = 0.01 p = .93 φ = 0.006 n = 258
	No	99	91	190	
Wet	Yes	37	43	80	χ^2^ (1) = 0.78 p = .38 φ = 0.05 n = 264
	No	96	88	184	
Cold	Yes	50	49	99	χ^2^ (1) = 0.37 p = .55 φ = 0.04 n = 264
	No	77	88	165	
Hot	Yes	34	33	67	χ^2^ (1) = 0.02 p = .89 φ = 0.01 n = 274
	No	103	104	207	

Note. φ = effect size. Respondents answering ‘Unsure’ were excluded.

**Table 11.** Comparison in response to damage visibility and helmet appearance before and after impact attenuation testing. Results for helmets dropped from 2 m

Conditioning	Damage visibility response	Drop height: 2 m			Chi-square tests of independence
		Before	After	Total	
Ambient	Yes	30	77	107	χ^2^ (1) = 34.86 p < .001 φ = 0.36 n = 269
	No	105	57	162	
Wet	Yes	35	72	107	χ^2^ (1) = 16.36 p < .001 φ = 0.25 n = 260
	No	89	64	153	
Cold	Yes	30	67	97	χ^2^ (1) = 23.10 p < .001 φ = 0.30 n = 266
	No	104	65	169	
Hot	Yes	53	90	143	χ^2^ (1) = 19.86 p < .001 φ = 0.27 n = 269
	No	81	45	126	

Note. φ = effect size. Respondents answering ‘Unsure’ were excluded.

All helmets dropped from 2 m along with the hot-conditioned helmet dropped from 0.34 m produced significantly higher rates in respondents answering ‘Yes’ as well as significantly lower rates in the response ‘No’ in association with their After photos as compared with their corresponding Before photos. No other significant associations were found among the remaining helmets dropped from 0.34 m or 0.42 m.

## 4. Discussion

This study describes a novel method in determining the relation between helmet structural damage and damage visibility as perceived by average consumers for impacts in line with concussion. Compared with previous studies where helmets were collected after real-world accidents, this study examined helmets immediately after impact attenuation testing for which impact severity is definitive (Smith et al. 1994; Ching et al. 1997). This approach reduces the possibility for error associated with more complex methods such as accident reconstruction. Another benefit to the chosen methodology is in the control over outside influences on damage visibility, as helmets were photographed within minutes of impact attenuation testing. Helmets collected through hospitalizations or manufacturer return policies, as in earlier studies, might unknowingly be affected before final inspection (Smith et al. 1994; Ching et al. 1997). Additional alterations to the helmets due to, for example, time in storage or transportation, are mitigated in this study.

### 4.1. Limitations and Assumptions

Concerning impact attenuation testing, the flat anvil used was assumed representative of most bicycle accident impact surfaces. Likewise, a natural response cannot be simulated by a rigid headform. Variation in a user’s head shape and helmet fit will affect the outcome of an impact and is unaccounted for by this study. Results of this study are specific to the chosen helmet model and are not transferable to other helmet models. Furthermore, angular effects on concussion threshold and helmet damage were not included in this study.

It is also important to address that polling results would have better represented reality if respondents had surveyed the helmets in person. Despite limitations to the polling method, the use of ‘Before’ photos as a control allowed for objective analysis.

### 4.2. Key Findings

While conditioning environment did not demonstrate a significant effect on the impact attenuation testing results, examining the individual results in Table 4, it was noted that for peak acceleration, HIC15, and SI, the cold-conditioned helmets consistently produced the highest values while the hot-conditioned helmets produced the lowest values in seven out of nine data points. This coincides with findings from earlier studies on EPS thermal behavior. EPS demonstrates a heightened strength with decrease in temperature and vice versa (Krundaeva et al. 2016). It is conceivable that such a reaction could lessen the protective cushioning abilities, resulting in, for example, higher values in peak acceleration, HIC15, and SI.

Analysis of the material testing results revealed that only the slope of the elastic buckling zone differed between helmets. This is of additional significance as this is where the dampening properties in EPS lie. A steeper elastic buckling slope equates to a more rapid dampening. In agreement with this, the 2 m drop height helmets produced the steepest slopes, indicating that their dampening abilities are the most diminished of the helmets tested. Furthermore, the linear regression results support the claim that elastic buckling slope increases with increased drop height. The conclusion can therefore be made that the EPS protective abilities diminish even after peak accelerations as low as 90–100 g.

Examination of the microscopic images reveals a noticeable decrease in conical crack propagation angle with increase in drop height. A decrease in this angle is directly related to an increase in Poisson’s ratio (Anongba 2019; Olivi-Tran et al. 2020). Poisson’s ratio and packing density are also closely connected; an increase in density coincides with an increase in Poisson’s ratio (Greaves et al. 2011). Therefore, a decrease in propagation angle correlates with an increase in density. This supports the finding that greater impacts effectively increase EPS density at the COI.

The lack of a significant difference in response for each of the 0.34 m and 0.42 m helmets, aside from the hot-conditioned 0.42 m helmet, implies that fewer people may notice damage in helmets sustaining concussion-threshold impacts of 90 to 100 g. These findings validate concerns that people may have difficulty noticing helmet damage through appearance alone, despite diminished protective abilities, especially for lesser impacts associated with concussion acceleration levels. These findings reiterate the recommendation on labelling by the CPSC and CSA that any accident-involved helmeted warrants a replacement, regardless of external appearance (C.S.A 1989; C.P.S.C 1998). With this in mind, it is recommended not to purchase helmets secondhand as helmet history cannot be verified through appearance alone.

### 4.3. Continued Study

Repetition of this study with a variety of helmets may account for variability between helmet models. While the findings of this study do not apply to all helmet models, possible trends discovered among multiple helmets would have greater transferability to unstudied helmets.

This study considered a distinct severity of brain injury – concussion. Moving forward, it would be of interest to examine damage visibility after incrementally more severe impacts to determine when damage is definitively visible to the average consumer.

## 5. Conclusions

For the chosen bicycle helmet model:

Helmet EPS liner damage increases linearly with impact severity. Altered material properties include an increase in density, elastic buckling slope, and densification slope at the COI. Out of which, the elastic buckling slope plays a significant role in EPS dampening ability.

Damage visibility is evident in helmets dropped from a 2-meter height. However, helmets impacted at the concussion-threshold drop heights of 0.34 m and 0.42 m do not present a notable difference in response to damage visibility.

Results of this study demonstrate how concussion-threshold impacts of 90 to 100 g are sufficient in negatively altering helmet protective abilities. Furthermore, the lack of a significant change in damage visibility between before and after photos may demonstrate consumers’ inability to recognize damage in helmets impacted at concussion-threshold accelerations. These findings support the claim that helmets can sustain damage without visual presentation.
